# Acknowledgement to Reviewers of *Life* in 2019

**DOI:** 10.3390/life10010004

**Published:** 2020-01-17

**Authors:** Life Editorial Office

**Affiliations:** MDPI, St. Alban-Anlage 66, 4052 Basel, Switzerland

The editorial team greatly appreciates the reviewers who have dedicated their considerable time and expertise to the journal’s rigorous editorial process over the past 12 months, regardless of whether the papers are finally published or not. In 2019, a total of 78 papers were published in the journal, with a median time to first decision of 15 days and a median time from submission to publication of 47 days. The editors would like to express their sincere gratitude to the following reviewers for their generous contribution in 2019:
Adam, ZacharyLu, YouAlbornoz, FranciscoLundin, DanielAmils, RicardoMagnuson, AnnAmundson, RonaldMalinge, Jean-MarcBains, WilliamMansy, SherefBaqué, MickaelMarkovitch, OmerBartlett, StuartMartínez-Romero, EsperanzaBasak, ShibajiMascetti, JoëlleBaum, David A.Matsuura, TomoakiBejaoui, SalmaMátyás, BenceBernhardt, Harold S.Mauksch, MichaelBonfanti, LucaMaurel, Marie-ChristineBorda, MichaelMaurel, Marie-ChristineBrack, AndreMichaelian, KaroBrack, AndréMizuuchi, RyoBraun, DieterMoran, JosephBroman, EliasMuchowska, Kamila BBuhse, ThomasMurugaiyan, JayaseelanBurton, Aaron S.Nanda, Sitansu SekharBurton, Zachary FNaraoka, HiroshiBurton, Zachary F.Newman, Stuart A.Cafferty, BrianNghe, PhilippeCallejón-Ferre, Ángel-JesúsNoffke, NoraChan, ClementNovikov, AndriyChatzitheodoridis, EliasNuevo, MichelClarke, PaulPark, ThomasClaudi, RiccardoPeacock-López, EnriqueCorenblit, DovPedreira-Segade, UlysseCronin, LeroyPinheiro, VitorCzárán, TamásPlasson, RaphaelDanger, GrégoirePolyansky, Anton A.Davank, VadimPopova, Alexandra A.De La Escosura, AndrésPotter-McIntyre, Sally L.De Los Santos, Emmanuel LorenzoPotvin-Trottier, LaurentDeamer, DavidPowner, MatthewDhakal, DipeshPreiner, MartinaDi Giulio, MassimoRabbow, ElkeDo, Chi-WaiRajamani, SudhaDołowy, KrzysztofRalser, MarkusDoolittle, Russell F.Ramirez, RamsesDreamer, DavidRamirez, RamsesDuvernay, FabriceRibó, Josep M.Egel, RichardRiek, RolandErastova, ValentinaRimmer, PaulFahrenbach, AlbertRimmer, PaulFerrante, AntonioRoberts, IreneFinster, KaiRosi, MarzioFiore, MicheleRoss, David S.Fologea, DanielRossi, Jean-ChristopheFornaro, TeresaRovner, AlexisForsythe, JayRuiz-Mirazo, KepaFrenkel-Pinter, MoranSaad, Nizar Y.Frossard, Jean-PierreSaha, RanajayFullmer, MatthewSahai, NitaFurukawa, YoshihiroSakamoto, KensakuGagnon, Jean-SebastienSeligmann, HervéGarner, CharlesShcherbakova, ViktoriaGeorgelin, ThomasShiomi, DaisukeGiner-Lamia, JoaquinShirt-Ediss, BenGrothe, HinrichSilvano, OnofriGrover, Martha ASmith, EricHao, JihuaSolovchenko, AlexeiHeckl, DirkSoumillion, PatriceHendrix, Donald A.Speijer, DaveHickman-Lewis, KeyronSpeth, Daan R.Higgs, PaulSponer, JuditHitzeroth, IngaStano, PasqualeHorváth, DezsöStern, Jennifer C.Horváth, JuditStevens, Adam H.Hsing, Yue-IeStrasdeit, HenryHuertas Romera, María JoséStrazewski, PeterIchihashi, NorikazuStrazewski, PeterIchihashi, NorikazuSuccurro, AntonellaIkehara, KenjiSurman, AndrewJafarpour, FarshidSzabo, ErikJakubowski, HieronimSzathmáry, EörsJheeta, SohanTakeuchi, NobutoJia, TonyTamarit, DanielJones, DaveyTamura, KojiJordan, SeanTaubner, Ruth-SophieJudit, ŠponerTaylor, AnnetteKaddour, HusseinTessera, MarcKakegawa, TakeshiTkachenko, AlexeiKarunakaran, SuneeshToner, JonathanKatin, Konstantin P.Torres, Adrian GabrielKawasaki, TsuneomiTrindle, CarlKee, TerenceVallée, YannickKeinath, MelissaVan Delden, SanderKieft, TomVasconcelos, F. A.Kippes, NestorVon Der Haar, TobiasKitadai, NorioWagner, NathanielKobayashi, KenseiWang, AnnaKönnyű, BalázsWang, AnnaKubyshkin, VladimirWeigand, WolfgangKun, ÁdámWills, Peter R.Kunnev, DimiterYabuta, HikaruLambert, Jean-FrancoisYang, JyisyLan, EthanYavin, EylonLancet, DoronYin, JohnLascelles, DesmondZagrovic, BojanLezcano Vega, María ÁngelesZanghellini, JürgenLi, YameiZobel, PatrickLingam, ManasviZrzavá, MagdaLopez Maury, LuisZuo, Ran

